# A Phylogenetic Host‐Range Index Reveals Ecological Constraints in Phage Specialisation and Virulence

**DOI:** 10.1111/mec.70052

**Published:** 2025-07-28

**Authors:** Clara Torres‐Barceló, Claudine Boyer, Julian R. Garneau, Stéphane Poussier, Isabelle Robène, Benoît Moury

**Affiliations:** ^1^ PHIM Plant Health Institute, Univ Montpellier, INRAE, CIRAD, Institut Agro, IRD Montpellier France; ^2^ CIRAD, UMR PVBMT St Pierre La Réunion France; ^3^ Department of Fundamental Microbiology University of Lausanne Lausanne Switzerland; ^4^ Université de la Réunion, UMR PVBMT St Pierre La Réunion France; ^5^ INRAE, Pathologie Végétale Montfavet France

**Keywords:** CRISPR, epidemiology, host range, phage, plant pathogen, *Ralstonia solanacearum*, virulence

## Abstract

Phages are typically known for having a limited host range, targeting particular strains within a bacterial species, but accurately measuring their specificity remains challenging. Factors like the genetic diversity or population dynamics of host bacteria are often disregarded despite their potential influence on phage specialisation and virulence. This study focuses on the *Ralstonia solanacearum* species complex (RSSC), which comprises genetically diverse bacteria responsible for a major plant disease. It uses a diversified collection of RSSC phages to develop new host‐range analysis methods and to test ecological and evolutionary hypotheses on phage host range. We introduce a new ‘phylogenetic host‐range index’ that employs an ecological diversity index to account for the genetic diversity of bacterial hosts, allowing systematic classification of phages along a continuum between specialists and generalists. We propose and provide evidence that generalist phages are more likely to be represented in CRISPR‐Cas immune system of bacteria than specialist phages. We explore the hypothesis that generalist phages might exhibit lower virulence than specialist ones due to potential evolutionary trade‐offs between host‐range breadth and virulence. Importantly, contrasted correlations between phage virulence and host range depend on the epidemiological context. A trade‐off was confirmed in a context of low bacterial diversity, but not in a context of higher bacterial diversity, where no apparent costs were detected for phages adapted to a wide range of hosts. This study highlights the need for genetic analyses in phage host range and of investigating ecological trade‐offs that could improve both fundamental phage knowledge and applications in biocontrol or therapy.

## 1 Introduction

Understanding the breadth and evolutionary constraints of phage host range is crucial for predicting microbial community dynamics and developing effective phage‐based therapies. A central question in phage ecology concerns whether specialisation imposes fitness trade‐offs, as a consequence of the difficulty to optimise infection on very diverse hosts (Visher and Boots 2020). This fundamental trade‐off between host range breadth and infection efficiency has broad implications for understanding pathogen evolution and could inform therapeutic strategies that exploit phage specificity.

The negative correlation between niche expansion and virulence has been proven across diverse host–parasite systems, from parasitic wasps and aphids, or microsporidians and shrimps, to herbivorous mites and plants, but we know little in phages and bacteria (Lievens et al. 2020; Schlenke et al. 2007; Skoracka et al. 2022). Experimental phage studies have shown that mutations enabling infection of new bacterial hosts frequently come at the cost of reduced fitness in the original host, yet evidence from natural populations remains limited (Duffy et al. 2006; Ferris et al. 2007).

However, this trade‐off is not universal. For instance, ‘trigger‐happy’ T‐even phages carry baseplate mutations that lower the activation threshold for tail‐fibre firing, resulting in both broader host range and faster infection (Riede et al. 1987). Similarly, in phage λ, Beardmore et al. (2023) showed that single mutations can confer dual benefits, increasing both infectivity and host range without incurring an apparent fitness cost. These counterexamples highlight that the specificity*–*virulence trade‐off may be context‐dependent, and that some phages can evolve towards broader infectivity without sacrificing performance, particularly under selective pressures from host diversity.

To resolve this uncertainty and develop predictive frameworks for phage host range, we need methodological approaches that can quantify both the breadth of infection and the evolutionary relationships among targeted hosts (Faria et al. 2013; French et al. 2023; Longdon et al. 2018). Despite the fundamental importance of characterising phage host range, current methodological approaches have significant limitations that hinder our understanding of specialisation patterns. In the past, most studies relied on qualitative spot tests (recording the occurrence or absence of bacterial lysis by the phage in a bacterial lawn) that could not distinguish between true resistance and abortive infections, potentially overestimating host range breadth (Daubie et al. 2022). Most recent studies use quantitative tests such as the efficiency of plating (EOP), which measures the phage titre (number of infectious phage particles) on a tested bacterium relative to a reference host (Mirzaei and Nilsson 2015). EOP is a valuable metric widely used in phage therapy to evaluate a phage's ability to multiply on target strains (Daubie et al. 2022; Green et al. 2017). Yet, while EOP effectively quantifies relative infectivity on specific hosts, it does not adequately capture the breadth of a phage's host range or the phylogenetic distances between targeted hosts.

Complementing these individual host‐phage measurements, researchers have increasingly turned to network‐based approaches that analyse the structure of host–parasite interaction matrices using metrics like nestedness and modularity (Flores et al. 2011; Weitz et al. 2013). Nestedness reflects hierarchical organisation in host–parasite interactions (e.g., resistance among bacteria and infection capacity among phages), while modularity describes the clustering of interactions into distinct groups. Still, network analysis describes properties of the entire phage–bacteria network without incorporating the phylogenetic context, limiting our ability to determine whether apparent specialisation reflects true evolutionary constraints or simply limited sampling of closely related hosts. Therefore, additional methods are needed to fully characterise host range diversity and ecological constraints.

Evidence from other pathogen systems suggests that phylogenetic relationships among hosts provide crucial constraints on infection patterns, offering a framework for predicting host range evolution (Desneux et al. 2012; Gilbert and Webb 2007; Guth et al. 2019). In plant and animal pathogen systems, the likelihood of successful infection is strongly constrained by host physiological similarities, which closely correlate with phylogenetic distance (Faria et al. 2013; French et al. 2023; Longdon et al. 2018). Taxonomic affiliation and explicit phylogenetic distances have been successfully used to predict infection success and quantify host breadth using diversity indices that incorporate phylogenetic structure (Poulin and Mouillot 2005). While this relationship has largely been explored in studies on plant and animal pathogens, it remains much less examined in phage‐bacteria models (Walsh et al. 2022). Phage–host range studies continue to focus predominantly on mechanistic determinants—such as receptor compatibility or host defence systems—while largely neglecting phylogenetic context (Beamud et al. 2023; Gaborieau et al. 2024; Howard‐Varona et al. 2018). This disconnect limits our ability to generalise about the evolutionary and ecological factors shaping phage host range and to provide a more integrative framework for understanding host specificity and its constraints.

To address these methodological and conceptual gaps, we developed an integrated approach that combines phylogenetic analysis with quantitative host range assessment to test fundamental hypotheses about phage specialisation. Specifically, we ask (1) Do phages exhibit phylogenetically structured host ranges, with infection success declining as host phylogenetic distance increases? (2) Is there evidence for specialisation–generalisation trade‐offs when host range is measured in a phylogenetically informed context? We test these hypotheses using phage–bacteria interaction network metrics and novel phylogenetically informed indices to characterise patterns of specialisation.

The *Ralstonia solanacearum* species complex (RSSC or *Ralstonia* spp.) provides an ideal system for testing these hypotheses due to its well‐characterised phylogenetic structure and significant agricultural importance. RSSC strains are responsible for bacterial wilt, one of the major bacterial diseases affecting *Solanaceae* crops, which include tomato, potato and aubergine, and 23 more botanical families, such as the *Musaceae* (banana), with devastating impacts on food security in tropical regions (Guji et al. 2019; Jiang et al. 2017). The RSSC has long been known for its high genetic and phenotypic diversity (Mansfield et al. 2012). RSSC strains were classified into three species and four major phylogenetic groups, named phylotypes: *R. pseudosolanacearum* including phylotypes I and III strains, *R. solanacearum* grouping phylotypes IIA and IIB strains and *R. syzygii* comprising phylotype IV strains (Mansfield et al. 2012; Prior et al. 2016; Safni et al. 2014). This hierarchical phylogenetic structure, in which each phylotype is further subdivided into multiple sequevars based on partial sequencing of the endoglucanase (*egl*) gene, provides the resolution necessary to test phylogenetic constraints on phage host range (Cellier et al. 2025; Fegan and Prior 2005; Poussier et al. 2000). Moreover, each sequevar comprises several haplotypes based on a multilocus variable number of tandem repeats (Rasoamanana et al. 2020).

Our study focuses on RSSC populations from Reunion and Mauritius in the South‐West Indian Ocean (SWIO), which exhibit contrasting patterns of genetic diversity that enhance our ability to test specialisation hypotheses. In Reunion, phylotype I dominates (70% of strains), with sequevar I‐31 comprising 94% of all strains, while sequevars I‐13, I‐33, IIA‐36, IIB‐1 and III‐19 occur at low frequencies (Yahiaoui et al. 2017). In contrast, Mauritius shows broader phylogenetic diversity within phylotype I (the only one currently present), with five sequevars (I‐14, I‐15, I‐18, I‐31 and I‐33) and lower dominance of sequevar I‐31 (19%) (Dookun et al. 2001; Cellier et al. 2023; Yahiaoui et al. 2017). This geographic variation in host diversity allows us to examine whether phage specialisation patterns are consistent across different host community structures.

Despite its genetic diversity and worldwide distribution, the interactions of RSSC with phages remain largely uncovered. An increasing number of phage genomes attacking *Ralstonia* spp. have been published (425 in the Inphared database, February 2025), although many (318) are prophage sequences and have not been isolated (Cook et al. 2021; Greenrod et al. 2022). Most studies focus on the characterisation of one or few phages and their biocontrol potential, showing promising results (Wang et al. 2019; Xavier et al. 2022). The host range of these phages, as well as their capacity to target the genetic and geographical variability of RSSC, is unknown. Previously described genetically diverse RSSC phages isolated in Mauritius and Reunion islands, together with the wide genetic diversity of their host, provide us with a unique opportunity to test ecological and evolutionary hypotheses about phage specialisation while implementing a new host‐range analysis methodology (Trotereau et al. 2021).

Finally, we explore CRISPR‐Cas systems as molecular records in the bacterial genome of historical phage–bacteria interactions to test our specialisation hypotheses from an evolutionary perspective (Meaden et al. 2022). Even though CRISPR‐Cas systems have not been proven to be the primary anti‐phage defence system in *Ralstonia* spp., 31% of their genomes harbour at least one (Xavier et al. 2018). Given that CRISPR‐Cas systems record past phage infections, we hypothesise that generalist phages will be more frequently represented in CRISPR‐Cas spacer databases than specialist phages. This prediction allows us to test whether phenotypically measured host range patterns are consistent with evolutionary signatures of phage–host interactions preserved in bacterial genomes.

## 2 Materials and Methods

### 2.1 Phages

The 23 *Ralstonia* spp. phages used in this study have been described elsewhere (Trotereau et al. 2021). Taxonomically, these 23 phages represent 13 new species and 7 new genera. This collection has morphologically diverse phages, comprising myoviruses, podoviruses and siphoviruses, and 17 have been determined as virulent and 6 as temperate phages. They share low genetic similarity between each other and with previously described phages (Trotereau et al. 2021). Their genomes correspond to *GenBank* accession numbers MT740725 to MT740747. The strain RUN3665 (sequevar I‐31) was used as the common host for subsequent phage amplification and titre standardisation of Reunion Island phages. Mauritian phages were amplified and standardised in three different host strains because no common host could be found in a host range pre‐screening analysis: RUN4407 (sequevar I‐15), RUN4833 (sequevar I‐33) and RUN5163 (sequevar I‐31). All phages were sequenced after the amplification.

### 2.2 Bacteria

RSSC strains and three strains of closely related species ( *Burkholderia cepacia* , *Ralstonia eutropha* and *Ralstonia pickettii* ), belonging to the collection of the ‘Pôle de Protection des Plantes’ in Reunion Island, were used for this study (Table S1). None of them were sampled at the same time or exact places as the phages. All inoculations and bacterial cultures were carried out in a semi‐selective Kelman culture medium containing triphenyltetrazolium chloride. Luria Bertani medium was used for non‐RSSC bacteria. All bacterial cultures were incubated at 28°C and liquid ones were agitated at 80 or 120 rpm, when including phages or not, respectively.

### 2.3 Host‐Range Assay

The bacterial strain panels used for phage host range testing were designed to represent the genetic diversity of RSSC found locally on each island, in the broader SWIO region and globally. Strains were selected based on local prevalence, genetic representativeness and inclusion of standard reference strains commonly used in the field (e.g., GMI1000). The island‐specific composition of each panel was necessary to capture the unique bacterial diversity present on each island, while the phylogenetically informed analytical approach allowed for meaningful comparison of phage specialisation patterns despite panel differences. The 10 Reunion phages were exposed to 52 RSSC and the 13 Mauritian phages to 63 RSSC, in addition to the three non‐RSSC strains ( *Burkholderia cepacia* , *Ralstonia eutropha* and *Ralstonia pickettii* ). Both panels shared 31 common strains that were representative of major phylotypes and sequevars found in the SWIO region, ensuring comparative analysis between islands. Both panels contained similar numbers of strains representative of RSSC genetic diversity across the three species and four phylotypes, with 5–7 strains from each sequevar IIA, IIB, III and IV (24 total for Reunion, 23 for Mauritius). However, phylotype I was intentionally overrepresented with 28 strains in the Reunion panel and 40 strains in the Mauritius panel, reflecting its epidemiological predominance. The Reunion panel included 23 local strains from Reunion, whereas the Mauritius panel included 27 local strains from Mauritius.

To assess the host range of each of the 23 phages, the phage titres were standardised to enable meaningful comparisons across samples. Due to technical constraints, the titre was 3.5 × 10^5^ pfu/mL for Mauritius and to 1 × 10^6^ pfu/mL for Reunion, as some Mauritian phages did not reach the higher concentration. A lower phage titre is indicative of a low frequency of spontaneous phage‐resistant bacterial mutants and is therefore suited for assessing host susceptibility (Hyman and Abedon 2010). Since calculations for phylogenetic host‐range index (PHRI; see below), virulence and other metrics are performed separately and with different panels of strains, we believe this approach does not introduce significant limitations. Phage infectivity was assessed by applying drops of 10 μL of serial 10‐fold dilutions, in triplicate, on the surface of the semi‐solid bacterial lawn of each strain. The phage load, that is, the number of phages in pfu/mL able to replicate on each strain, also referred to as the infectious population size in this work, was recorded using the most appropriate dilution.

For the Reunion phage–bacteria data set, we calculated the EOP classic metric, as the log10 transformed phage load on a tested bacterium relative to a reference host. All Reunion phages were propagated on the same bacterial strain, allowing more reliable cross‐comparisons than for Mauritian ones. The number of strains with an EOP greater than 0.1 was used as a binary measure of host range breadth, while the mean EOP across all tested strains was calculated as a general estimate of average infectivity.

### 2.4 Phage Phylogenetic Host‐Range Index

A phylogenetic host‐range index (PHRI) was calculated for each phage as a proxy for the diversity of bacterial strains it can attack and how efficiently it replicates in them. It accounts for the number of bacterial strains targeted, the relative phage load (pfu/mL) on each strain (i.e., evenness) and the phylogenetic distance between bacterial hosts (Alberdi and Gilbert 2019; Chao et al. 2010). The PHRI is based on the phylogenetic abundance‐based Allen index that incorporates relative abundance and species richness, as calculated with the ‘hilldiv’ R package (Allen et al. 2009). Compared to other indices, Allen's index maximises the importance of highly distinct species, which we consider essential to characterise a phage host range (the maximum diversity of bacteria they are able to infect) (Allen et al. 2009).

The data used for this analysis were the quantitative matrix of phage–bacteria host range interactions (phage load on each strain) and a phylogenetic tree of the bacterial strains tested. The phylogenetic distance between bacterial hosts was calculated using the phylogenetic tree of the RSSC endoglucanase (*egl*) gene, a well‐established molecular marker for studying RSSC diversity and classification (Cellier et al. 2025). The *egl* gene has been widely used to assign RSSC strains into distinct phylotypes and sequevars, offering high resolution for distinguishing closely related strains and serving as a standard tool in RSSC phylogenetics (Cellier et al. 2023; Poussier et al. 2000). Due to the different RSSC strains used and the different standardised phage titre of Mauritius and Reunion phages, the PHRI was calculated separately for phages of each geographical origin.

### 2.5 Genomic Analyses

To investigate the genetic factors underlying differences in host‐range breadth among closely related phages, all phages were re‐annotated using PROKKA (Seemann 2014), with a priority annotation using the PHASTER protein database (version Dec 22, 2020) (Arndt et al. 2016). For each pair of phages compared, predicted phage proteomes were analysed pairwise using Roary with a 90% protein identity threshold to identify shared and unique proteins (Page et al. 2015). The resulting presence–absence matrices of proteins can be found in Table S2.

To identify potential host interactions and immunity relationships, we screened the 23 phage genomes from our internal collection for matches to CRISPR spacers derived from *Ralstonia* spp. and related bacteria. We used the CrisprOpenDB database that compiles CRISPR spacers from all bacterial genomes available in the NCBI database as of March 23, 2020, encompassing 580,383 bacterial genomes in total, including 38 genomes of *Ralstonia solanacearum* (Dion et al. 2021). From these 38 genomes, a total of 1988 unique CRISPR spacers were identified. The CrisprOpenDB command‐line host prediction tool (https://github.com/edzuf/CrisprOpenDB) was installed locally to search for spacer matches in the 23 phage genomes. Searches were performed using the recommended mismatch threshold of 2, with the following command:

$pythonCL_Interface.py‐‐mismatch2‐‐aligner blast‐‐table‐iPhage_Genome.fasta

The resulting output files were manually consolidated (Table S3). We quantified the number of matches per phage genome, counting only one match per distinct *R. solanacearum* strain and recorded the corresponding bacterial genome sources.

### 2.6 Phage–Bacteria Network Structure Analysis

To identify non‐random patterns in phage–bacteria interactions, we analysed the nestedness and modularity of the two interaction matrices from Mauritius and Reunion. We estimated the nestedness and modularity scores of the two phage–bacteria matrices using various algorithms implemented in the R packages ‘bipartite’ and ‘igraph’, as described in Moury et al. (2021). In addition, we incorporated the DIRTLPAwb+ and QuanBiMo modularity algorithms implemented in the ‘bipartite’ package to improve modularity detection (Beckett 2016; Dormann and Strauss 2014). Only the subset of bacterial strains where at least one phage could replicate was used in this analysis (13 Mauritian phages confronted to 33 RSSC strains and 10 Reunion phages confronted to 20 RSSC strains). The principles of nestedness and modularity analyses are available in Weitz et al. (2013). Prior to these analyses, in each matrix separately, phage load values (titre of each phage measured on each bacterial strain) were binned into 10 intervals of equal size, as described in Moury et al. (2021). Finally, the statistical significance of nestedness and modularity was assessed by comparing the nestedness and modularity scores of the actual matrices with those of 100 random matrices generated according to various null models. More details can be found as Supplementary Methods.

### 2.7 Phage Host‐Range Phylogenetic Signal

To assess whether phage host range shows a phylogenetic signal (i.e., whether phages tend to infect genetically related bacterial strains more than distant ones) we calculated the phylogenetic signal for each phage. Using the phage load matrix and corresponding bacterial phylogenetic trees (the same data as for PHRI), we applied the Bayesian mixed model approach implemented in the *MCMCglmm* R package.

### 2.8 Phage Virulence

To evaluate the virulence of phages, we measured the inhibitory effect on bacterial growth of 10 Reunion phages and 11 Mauritian phages (Hennie and Hyacinthe phages were not included due to their extreme specificity). The three most abundant bacterial strains on each island were used for each set of phages, namely RUN4407 (sequevar I‐15), RUN4833 (sequevar I‐33) and RUN5163 (sequevar I‐31) for Mauritius phages, and three different haplotypes of sequevar I‐31 (RUN3665, RUN3014 and RUN3692) for Reunion phages. First, bacteria were incubated overnight and the optical density at 600 nm (OD600) homogenised to 0.1 (10^8^ cfu/mL) in the morning. Phages were tested against bacteria at 10^6^ pfu/mL, corresponding to a phage:bacteria ratio (MOI or Multiplicity of Infection) of 0.01, in a final volume of 200 μL. The chosen phage dose represented an intermediate dose capable of reducing bacterial growth substantially but not completely. Three replicates of growth curves were recorded by measuring the OD_600_ every 15 min over 24 h. All experiments were done using the plate spectrophotometer Bioscreen (Oy Growth Curves Ab Ltd., Finland). Positive controls (phage‐free bacterial cultures) and negative controls (bacteria‐free) were included in each plate. The experiments were performed twice, using two independent plates.

To analyse the data, an average of all OD_600_ measurements over time of each replicate culture was calculated. The mean blank value (negative control without bacteria and phages) was subtracted from all the mean OD_600_ values. The inhibitory effect of phages on bacterial growth was calculated as the percent average growth difference between treatments (phage‐infected) and positive controls (uninfected):

$$\begin{array}{c} \text{Phage virulence}\;\left(\%\right)\\ =100-\left(\left({\text{bacterial growth with phage}}^{*}\;100\right)/\text{bacterial growth in positive control}\right) \end{array}$$

The maximum inhibitory capacity of a phage was selected as the highest three replicate measurements of inhibition on a given bacterial strain. This value was used in particular analysis, as detailed in the statistics section (Section 2).

### 2.9 Correlation Analysis Between Phage Load and Host Range

To test for a statistically significant relationship between phage load and host range (PHRI) while accounting for their non‐independence, we calculated the average infectious population size (phage load) for each phage, considering only bacterial strains where phage presence was detected (excluding non‐sensitive strains). As the calculation of the phage PHRI is partly based on their load estimates, there is a risk of spurious correlation when analysing the correlation between the non‐independent ‘PHRI’ and ‘phage load’ variables (Brett 2004). To circumvent this pitfall, we defined an ad hoc null hypothesis based on the distribution of the coefficients of correlation obtained by using 1000 random permutations, as suggested previously by Jackson and Somers (1991). For each permutation, the phage load values were first randomly permutated across bacterial strains. Then, we calculated the PHRI of this permutated data set and we estimated the Pearson's correlation coefficient between PHRI and phage load. The comparison between the distribution of the 1000 Pearson's correlation coefficients obtained from random permutations and the correlation coefficient from the actual data allowed us to rigorously test the linear dependence between phage load and PHRI.

### 2.10 Additional Statistical Analyses

To test the association between phage host range (PHRI) and the number of unique CRISPR spacers in bacterial hosts, we fitted a generalised linear model (GLM) with a quasi‐Poisson distribution and a log link function. To investigate virulence differences among phages across bacterial strains and between the two islands of origin, we fitted a linear mixed effects model including ‘plate’ as a random effect.

To examine the influence of taxonomy (species and genus), life cycle (temperate vs. virulent) and geographical origin (Reunion vs. Mauritius) on phage traits, we applied non‐parametric Kruskal–Wallis tests. The dependent variables tested were PHRI, phage host‐range phylogenetic signal, maximum inhibitory capacity and phage load. Associations between phage traits (PHRI, host‐range phylogenetic signal, maximum inhibitory capacity, phage load) and genomic features (genome size, GC content, protein content) were evaluated using Spearman correlation analyses (Trotereau et al. 2021).

We tested homogeneity of variances in PHRI between islands with Levene's test, and for variables with more than two categories, significant differences between means were identified using Tukey's post hoc multiple comparisons test. All statistical analyses were performed using R version 3.5.1 (http://cran.r‐project.org/). Model assumptions were tested by visually exploring model residuals.

## 3 Results

### 3.1 The Host Range of *Ralstonia* spp. Phages Extends From Extreme Specialists to Wide Generalists

**FIGURE 1:**
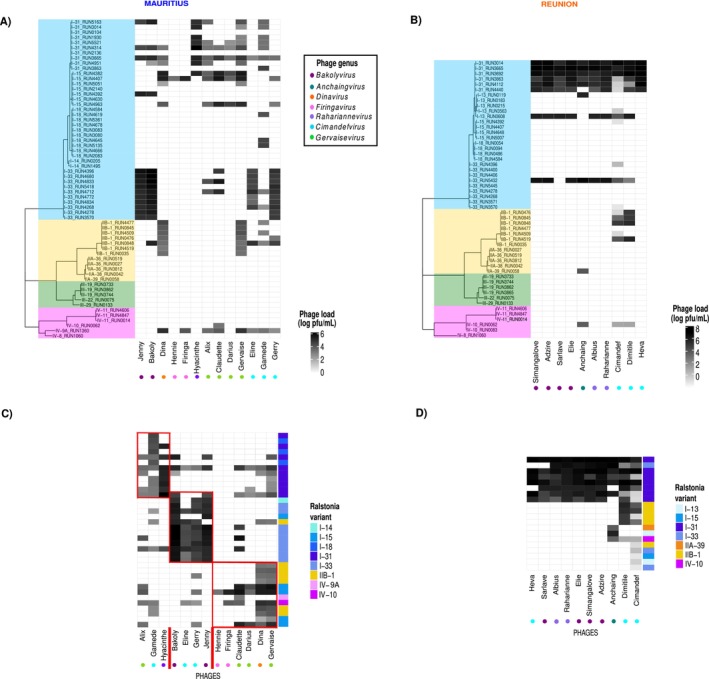
Quantitative heatmaps representing the host range of phages. The load or amount of progeny (pfu/mL) produced by each phage on each bacterial strain is indicated by a grey gradient code. Phage genera are depicted at the bottom with colour dots and bacterial phylotypes are indicated in different colours. Panels (A) and (B) show phylogenetic heatmaps of 63 RSSC strains against 13 Mauritian phages, and 52 RSSC strains against 10 Reunion phages, respectively. Panels (C) and (D) illustrate quantitative heatmaps with the network analysis of the host range of phages. Panel (C) shows infection profiles for 33 RSSC strains against 13 Mauritian phages, while Panel (D) represents 20 RSSC strains against 10 Reunion phages. Only bacteria infected by at least one phage were included in the analysis. The modular structure of Mauritius data set, with the three modules delimited by red lines (C), and the nested structure in Reunion data set (D) are shown.

Considering the high genetic diversity of the RSSC, we examined if phages were adapted only to the local diversity of RSSC or if their host range extended beyond. To answer this question, we confronted 13 Mauritius and 10 Reunion phages to local and international strains of the host species. None of the phages targeted the three non‐RSSC species tested, supporting a restricted host range of phages beyond the RSSC. All of them targeted strains from phylotype I, the most prevalent (87%) in the SWIO, reflecting their adaptation to the local diversity (Yahiaoui et al. 2017) (Figure 1A,B). Eleven phages (7/10 from Reunion, 4/13 from Mauritius) replicated only on strains belonging to this phylotype, and 12 others (3/10 from Reunion, 9/13 from Mauritius) replicated in strains from this phylotype as well as from phylotypes II and IV (Figure 1). Two RSSC strains from phylotype IV were isolated in 2017 in Mauritius, but this phylotype has never been recorded in Reunion (Yahiaoui et al. 2017). The replication of phages from both islands on phylotype IV bacteria can be explained by a genetic convergence of phage replication mechanisms or by the existence of conserved receptors in bacteria from phylotypes IV and I and/or phylotypes II and I. No phage could replicate in the tested strains from phylotype III, represented only in Reunion at a low frequency (8%) and on rare plant hosts (Yahiaoui et al. 2017). No strains from phylotype II have been recorded in Mauritius since 2010 but they are currently present in Reunion (22%) (Khoodoo et al. 2010). These results suggest that the epidemiological history of bacteria in the islands could be reflected, at least partially, in the phages host range, with many Mauritian phages preserving their capacity to target the now disappeared phylotype II.

At the sequevar level, all phages but two Mauritian ones (Hennie and Firinga) target at least one strain of sequevar I‐31, the most dominant genetic variant of the RSSC in Reunion (Figure 1A). Seven out of 10 Reunion phages target only strains of sequevar I‐31, suggesting a strong specialisation (Figure 1B). Most Mauritian phages target a relatively wide diversity of phylotype I sequevars, namely I‐14, I‐15 and I‐33, besides I‐31. The overall load (pfu/mL) of phages of Reunion in the tested strains was higher than that of Mauritius phages (*χ* ^2^ = 12.062, df = 1, *p* < 0.001), suggesting that the load of Mauritius phages could be reduced as a trade‐off of their generalist host range, even considering initial titre variations between Mauritius and Reunion phage preparations.

**FIGURE 2:**
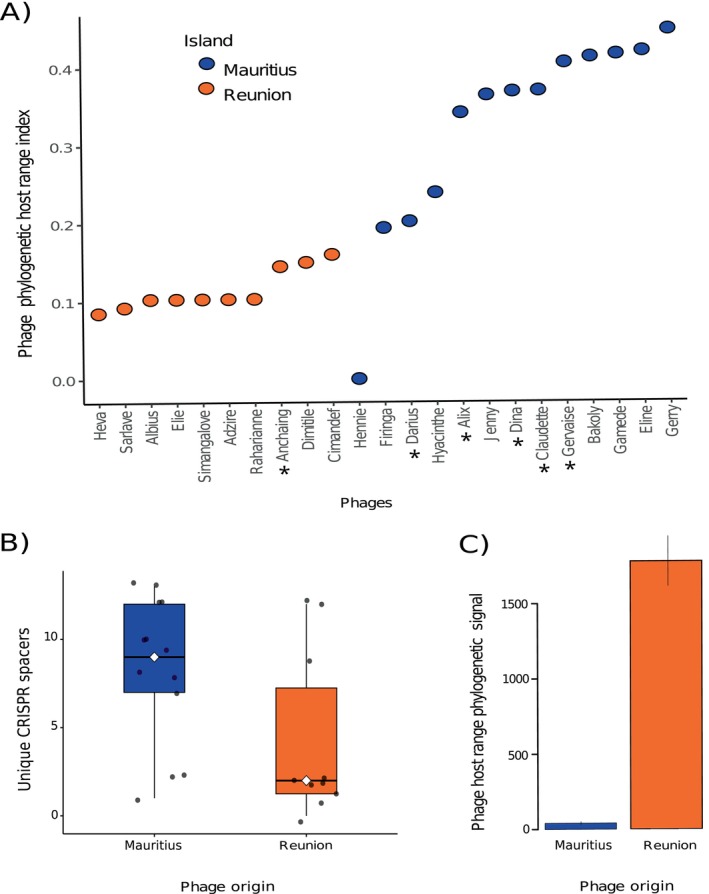
(A) Phage phylogenetic host‐range diversity index, where higher index values indicate that phages are able to infect and replicate better on a broader range of genetically diverse bacterial strains. Asterisks next to phage names indicate a temperate lifestyle. (C) Phylogenetic signal of host strain susceptibility, measuring the degree to which the strains targeted by *Ralstonia* spp. phages are phylogenetically related, depending on the island of origin of each phage. Higher values indicate stronger phylogenetic clustering. Data are average phylogenetic signal and standard errors. Both (A) and (C) data sets are based on 13 Mauritian phages confronted to 33 RSSC strains, and 10 Reunion phages confronted to 20 RSSC strains. (B) Distribution of unique CRISPR spacer counts in *Ralstonia solanacearum* genomes matching the 13 Mauritian and 10 Reunion phages, grouped by island. White diamonds and line indicate the median for each group.

We investigated whether phage host ranges follow phylogenetic patterns, whereby infection success decreases as phylogenetic distance from the host increases. We incorporated phylogenetic approaches to test host range patterns, adjusting for differences in bacterial panels between islands, which reflected the specific bacterial diversity on each. This phylogenetic framework provided a quantitative and more complete measure of phage host range while controlling for the over‐representation of phylotype I strains in our data set. We used Allen's phylogenetic diversity index, which accounts for the number of bacterial strains targeted (i.e., infected), the relative phage load (pfu/mL) on each strain (i.e., evenness), and the phylogenetic distance between bacterial hosts (phylogenetic tree of the RSSC *egl* gene) (Allen et al. 2009). The PHRI was significantly higher for Mauritian than Reunion phages (*χ* ^2^ = 11.635, df = 1, *p* < 0.001), meaning that they can target and replicate better on more strains that are genetically more distant (Figure 2A). The PHRI of Mauritian phages has also a larger variation than that of Reunion phages (Levene's test *F*_1,21_ = 4.3355, *p* = 0.0497), ranging from extreme specialist (Hennie) to generalist phages (Eline and Gerry) (Figure 2A). The PHRI of Reunion phages is far more homogeneous and smaller overall, reflecting the specialisation of most of them on closely related strains from sequevar I‐31. Surprisingly, the six temperate phages have a relatively high PHRI, as opposed to the trend found in other studies (de Sousa et al. 2021). In summary, we quantified the host range of phages integrating the host genetic information and observed important differences related to their geographical origin and to the bacterial diversity in these areas. The host range of phages is adapted to the local bacteria, but some generalist phages are able to target distantly related strains.

To evaluate how PHRI compares with traditional metrics, we calculated both EOP and binary infection scores (*N*, number of strains infected) for the Reunion phage data set (Table S4). While all three metrics identified *Heva* as the most specialist and *Cimandef* and *Dimitile* among the most generalist phages, intermediate cases showed disagreement between metrics. The range of values was highest for *N*, followed by EOP and PHRI. The larger range observed for *N* and EOP suggests that these metrics may overestimate host range by including marginal or low‐efficiency infections. In contrast, PHRI integrates the number of infections (*N*), their efficiency (EOP) and the phylogenetic relationships among hosts, allowing comparison of host range breadth across phages.

### 3.2 Generalist Phages Are More Frequently Represented Among CRISPR‐Cas Spacers Than Specialists

The genomic novelty of these phages limits the understanding of the encoded gene functions and their association with the host‐range analysis. In fact, the annotation of the genomes of all 23 viruses is partial, with more than 60% of proteins having unknown functions, and only a few tail fibre genes clearly determined (which usually contain the receptor‐binding proteins). In order to explore the genetic basis of host‐range breadth, we compared the genome of six pairs of phage species from the same genus (Table S2) with contrasting host ranges (e.g., Heva vs. Cimandef, two species from the genus *Cimandefvirus* that are the least and most generalist phages of Reunion, respectively), using methods including re‐annotation with PROKKA and protein clustering with Roary (see Section 2). The analysis did not reveal radical differences in gene content or gene length, except for the presence of more annotated endonucleases in phages with narrower host ranges (Table S2). For instance, phages Heva and Simangalove, both with a narrow host range, possess a CRISPR‐associated endonuclease Cas9. Additional functional annotation and receptor‐binding assays would be necessary to understand the relevance of these findings.

Next, we examined bacterial genomic data. CRISPR‐Cas spacers record historical phage–bacteria interactions, and we propose that generalist phages, by infecting a broader range of hosts, are more likely to be targeted and thus more frequently represented in CRISPR arrays than specialist phages. While the genome sequences of the RSSC strains used in this study are not available, we searched for matches to the 23 phage genomes in CRISPR‐Cas spacers sequences from publicly available bacterial databases, including 38 *R. solanacearum* strains (Table S3) (Dion et al. 2021). We identified 250 spacer matches allowing up to two mismatches against 22 of our phages (all but Anchaing). Of these, 233 spacers originated from *R. solanacearum* and were distributed across 20 of the 38 genomes. The remaining 17 spacers, which came from other bacterial species, were excluded from further analysis. All matches were identified using stringent criteria, ensuring high‐confidence assignments despite the limited number of informative genomes. Across all samples, no significant overall association was detected between PHRI and CRISPR content (*β* = −0.99, *p* = 0.46). However, bacterial CRISPR spacer counts matching phages were significantly lower for phages from Reunion compared to those from Mauritius (*β* = −3.21, *p* = 0.031) (Figure 2B). These findings suggest that Mauritian phages may have more frequently encountered bacterial hosts harbouring CRISPR‐Cas systems, consistent with their broad host ranges (Figure 2A). Further work is needed to disentangle whether CRISPR targeting reflects generalism per se or correlates with other phage traits that influence encounter rates.

### 3.3 Bacteria–Phage Infection Matrices Are Both Modular and Nested

To complete the host‐range analysis, we were interested in the structure of the phage–bacteria infection matrices and the possible identification of modules, composed of bacterial strains and phage species interacting specifically. In light of the different normalisations of titres and panels of bacteria tested, separate network analyses were performed for Mauritius and Reunion phages. A strong modularity pattern was detected in the Mauritius matrix, with relatively high modularity values (> 0.35 on a scale spanning usually from −0.5 to 1, for all algorithms except *spinglass*) and a high statistical significance (*p* value < 0.01) with all algorithms and all null models (Table 1, Figure 1C). The minimum modularity score of the Mauritius matrix was 6%–28% (mean 20%) higher than the maximum modularity score of matrices simulated following null models, except for null model B, which is excessively conservative (‘effect sizes’ in Table 1; Moury et al. 2021). A weak nested pattern was also detected, especially with the most efficient *WINE* algorithm and the most efficient C1 + R1 and C2 + R2 null models (Table 1), meaning that there is a gradient of specialisation and generalism among phages. The analysis determined three modules in the Mauritian phage–bacteria matrix (Figure 1C).

**TABLE 1:** Analysis of nestedness and modularity of phage–bacteria interaction matrices.

Matrix	Analysis	Algorithm	Score^a^	Null model^b^						
				B	N	C1	R1	S	C2	R2
Reunion	Nestedness	wNODF	0.36	1.00 ^c^	< 0.01 (1.20)	< 0.01 (1.22)	1.00	< 0.01 (1.35)	< 0.01 (2.00)	< 0.01 (1.14)
		WINE	0.72	< 0.01 (1.30)	< 0.01 (3.58)	< 0.01 (6.56)	< 0.01 (1.23)	< 0.01 (2.78)	< 0.01 (6.71)	< 0.01 (1.11)
	Modularity	spinglass	0.08	< 0.01 (1.14)	< 0.01 (1.10)	< 0.01 (1.17)	< 0.01 (1.33)	< 0.01 (1.08)	< 0.01 (1.10)	< 0.01 (1.17)
		edge betweenness	0.09	< 0.01 (1.57)	1.00	1.00	0.05	1.00	1.00	< 0.01 (1.06)
		fast greedy	0.15	< 0.01 (1.28)	1.00	1.00	0.13	1.00	1.00	< 0.01 (1.17)
		leading eigenvector	0.15	< 0.01 (1.40)	0.97	0.99	< 0.01 (1.03)	0.96	0.99	< 0.01 (1.20)
		louvain	0.15	< 0.01 (1.19)	1.00	1.00	0.66	1.00	1.00	< 0.01 (1.11)
		DIRTLPAwb+	0.16	< 0.01 (1.33)	1.00	1.00	0.15	1.00	1.00	< 0.01 (1.10)
Mauritius	Nestedness	wNODF	0.16	1.00	0.12	0.61	0.90	0.09	0.02	0.02
		WINE	0.39	1.00	< 0.01 (1.58)	< 0.01 (1.39)	< 0.01 (1.54)	< 0.01 (2.28)	< 0.01 (1.32)	< 0.01 (1.79)
	Modularity	spinglass	0.10	< 0.01 (1.08)	< 0.01 (1.23)	< 0.01 (1.28)	< 0.01 (1.23)	< 0.01 (1.17)	< 0.01 (1.16)	< 0.01 (1.23)
		edge betweenness	0.37	< 0.01 (5.87)	< 0.01 (1.22)	< 0.01 (1.36)	< 0.01 (1.29)	< 0.01 (1.25)	< 0.01 (1.41)	< 0.01 (1.32)
		fast greedy	0.37	< 0.01 (2.65)	< 0.01 (1.10)	< 0.01 (1.17)	< 0.01 (1.14)	< 0.01 (1.17)	< 0.01 (1.27)	< 0.01 (1.18)
		leading eigenvector	0.36	< 0.01 (3.07)	< 0.01 (1.10)	< 0.01 (1.15)	< 0.01 (1.18)	< 0.01 (1.15)	< 0.01 (1.23)	< 0.01 (1.30)
		louvain	0.39	< 0.01 (2.61)	< 0.01 (1.06)	< 0.01 (1.10)	< 0.01 (1.12)	< 0.01 (1.11)	< 0.01 (1.22)	< 0.01 (1.19)
		DIRTLPAwb+	0.39	< 0.01 (2.76)	< 0.01 (1.08)	< 0.01 (1.13)	< 0.01 (1.16)	< 0.01 (1.18)	< 0.01 (1.24)	< 0.01 (1.23)

*Note:* ‘< 0.01’ indicates that none of the 100 simulations had a strictly higher nestedness (or modularity) score than that of the actual matrix. In these cases, we indicated an ‘effect size’ between parentheses, calculated as the minimum modularity (or nestedness) score of 100 estimations based on the actual matrix divided by the maximum modularity (or nestedness) score of 100 matrices simulated following the corresponding null model.

^a^ Mean of 100 estimates. The score varies usually from 0 to 1 for nestedness and from −0.5 to 1 for modularity.

^b^ See the Section 2 and Moury et al. (2021) for details of the null models.

^c^ Nestedness or modularity significance: the probability value (*p* value) indicates the frequency of null‐model matrices among 100 simulations showing a strictly higher nestedness (or modularity) score than that of the actual matrix. *p* Values ≤ 0.05 (significant nestedness or modularity) are on grey cells, and *p* values > 0.95 (significant anti‐nestedness or anti‐modularity) are in white on black cells.

Module 1 was characterised by three viruses from different genera, *Gervaisevirus Claudette* (clone Alix), *Cimandefvirus Gamede* and *Rahariannevirus Raharianne* (clone Hyacinthe), and all bacteria from sequevars I‐31 and I‐18. *Cimandefvirus* and *Rahariannevirus* phage genera are widely represented in Reunion and thus specialised in sequevar I‐31, suggesting that they originated there and dispersed later to Mauritius. Alternatively, they may have historically been present in Mauritius but have become less prevalent due to a decline in the abundance of their I‐31 hosts. Module 2 included four phages from two genera with a wide host range according to the PHRI analysis, *Bakolyvirus* and *Cimandefvirus*, and all the strains from sequevar I‐33 and the only one from I‐14. These phages belong to genera also found in Reunion but, especially those from the genus *Bakolyvirus*, were extensively sampled in Mauritius Island (Trotereau et al. 2021). This implies that they could have first appeared in Mauritius and expanded later to Reunion Island, the opposite scenario described for the phages of Module 1, or their prevalence could be adapted to changes in host population dynamics. Module 3 integrated six Mauritian phages from three genera and bacteria from three phylotypes of RSSC (I, II and IV). The distinctive trait is that all the phages target strains of sequevar I‐15, and most can target strains from the distant RSSC phylotype IV (Trotereau et al. 2021). In hindsight, modules are mostly associated with bacterial taxonomy. In contrast, some phages from the same genera and even the same species belong to different modules (Figure 1C). This suggests a fast evolution of some phage genes, probably tail fibres recognising bacterial receptors, but not at a complete genomic level. This is not surprising, taking into account the high potential of mutation and recombination of phages (i.e., the genetic mosaicism of phages) (de Sousa et al. 2021; Pal et al. 2007). The three modules contained phages with different PHRI ranges.

The phage–bacteria infection matrix from Reunion had a different structure. With the most efficient *WINE* algorithm, a high nestedness score was estimated (0.72), and all null models revealed a significant pattern (*p* value < 0.01; Table 1), suggesting that, in spite of the homogeneous PHRI, there were both relatively specialist and generalist phages (Figure 1D). The nestedness effect size varied greatly between null models, the minimum score of the Reunion matrix being 11%–571% (mean 332%) higher than the maximum nestedness score of matrices simulated following null models (Table 1). Phages Anchaing, Dimitile and Cimandef were the relatively generalist ones, whereas Heva was the most specialised, as in the previous PHRI analysis. A weak (0.08) but significant (*p*‐value < 0.01) modularity score was also detected with the *spinglass* algorithm for all null models (Table 1). Depending on null models, the minimum score of the Reunion matrix was from 8% to 33% (mean 16%) higher than the maximum modularity score of simulated matrices (Table 1). The five other modularity algorithms did not detect any significant modularity with the most efficient C1 + R1 or C2 + R2 null models. Similar to Mauritian phages, phage taxonomy was not always associated with the host‐range pattern, suggesting that adaptation of phages to the local bacteria is the most important explanatory variable. The nested structure revealed the most sensitive bacteria, namely sequevar I‐31, and the more resistant ones, sequevars I‐13 and I‐15. Interestingly, the PHRI values for Reunion phages perfectly mirrored the ascending order of generalism revealed by the matrix structure.

### 3.4 Phylogenetic Signal of Bacterial Hosts and Bacterial Sensitivity Differ Between Phages From the Two Islands

To test if the phage host‐range patterns detected were associated with a phylogenetic signal among the targeted bacteria, we calculated the genetic divergence of the strains infected by each phage. The phylogenetic signal of Mauritian phages is significantly lower than that of Reunion phages (*χ* ^2^ = 11.635, df = 1, *p* < 0.001), meaning that the strains they target are much less phylogenetically related (Figure 2C). This implies that similar molecular interactions may be at play in genetically distinct bacteria, suggesting analogous phage receptors or defence systems. The phylogenetic signal test supports the results obtained with the higher PHRI (generalism) for Mauritian phages. The high phylogenetic signal of the bacteria targeted by Reunion phages corroborates that bacterial epidemiology is likely the most determinant factor of phage host range. Differences in the genetic diversity of the tested phages are not involved in the host range measurements, as it is similarly high for Reunion (four genera and five species) and Mauritius (six genera and eight species) (Trotereau et al. 2021).

The relationships between the breadth of bacterial resistance (i.e., the number of phages a strain can resist) and the efficiency of resistance against each phage can reveal important evolutionary interactions, such as trade‐offs (e.g., broad but less effective resistance) or cases of non‐costly cross‐resistance (Wright et al. 2018, 2019). We propose that these interactions depend on the ecology and genetic variation of both bacterial hosts and phages. For a highly diverse host like RSSC, the trade‐off hypothesis appeared particularly plausible. We therefore analysed, among bacterial strains, the correlations between the resistance spectrum, that is, the number of phages that did not replicate in a given bacterial strain, and the average sensitivity to phages (excluding phages that did not replicate). For the Mauritius bacteria panel, no significant relationship was observed between resistance spectrum and sensitivity (Pearson's *r* = −0.155; *p*‐value = 0.37; Figure S1A). In contrast, for the Reunion bacteria panel, Pearson's coefficient of correlation was significantly negative (*r* = −0.695; *p* value = 6.7e‐04; Figure S1B) and hence the stronger the resistance of bacteria, the broader their resistance spectrum, contrary to the trade‐off expectations. According to these results, bacteria exposed to multiple phages did not exhibit a detectable resistance cost, consistent with previous observations (Wright et al. 2019). Additionally, bacterial resistance patterns aligned with the host range of the phages, suggesting distinct resistance profiles among bacterial variants from Reunion and Mauritius.

### 3.5 Phages Efficiently Inhibit Local Bacteria

**FIGURE 3:**
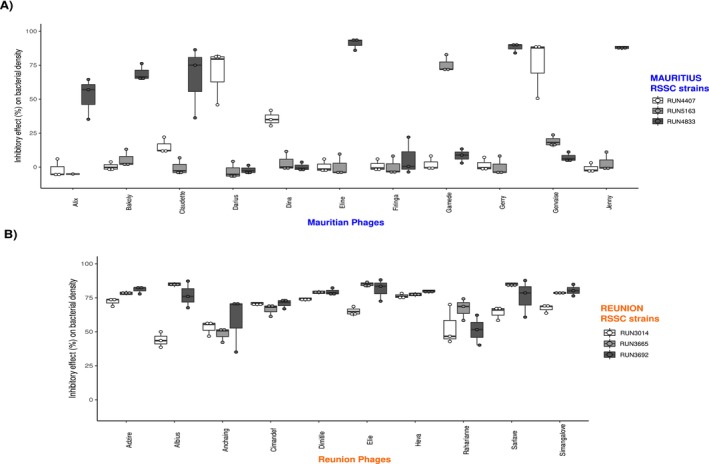
Virulence of phages measured as their inhibitory effect on bacterial growth, that is, difference in mean bacterial density (OD_600_) over 24 h in the presence and absence of phages. Eleven phages from Mauritius (A) and 10 from Reunion (B) were analysed using the three most abundant RSSC strains for each island. Data are average phage virulence and standard errors.

The bacterial inhibition efficacy or phage virulence against the three most abundant RSSC strains was, on average, lower for the phages from Mauritius (23% inhibitory effect) compared to those from Reunion (71% inhibitory effect) (*χ* ^2^ = 403.66, df = 1, *p* < 0.001) (Figure 3). However, this could be explained by the fact that the three strains tested for Reunion phages were genetically more similar (three haplotypes from sequevar I‐31) than those tested for the Mauritian phages (three different sequevars). Phage‐induced inhibition or average fitness cost suffered upon phage attack in Mauritian RSSC variants differed significantly between sequevars I‐33 (43%), I‐15 (18%) and I‐31 (9%) (*χ* ^2^ = 51.95, df = 2, *p* < 0.001), but no significant differences were found for Reunion strains (*χ* ^2^ = 1.3086, df = 2, *p* = 0.5198) (Figure 3). Also, the virulence of Mauritian phages was more variable, with some phages specialising in inhibiting efficiently only one of the three bacteria tested.

All but one of the tested Mauritian phages inhibited over 35% of bacterial growth in at least one strain, but exhibited extremely low virulence against two others, suggesting that they may be specialised on other untested host strains (Figure 3A). Most Reunion phages efficiently inhibited the three tested bacterial strains, with Anchaing (temperate) and Raharianne (virulent) phages having the lowest virulence (50%) (Figure 3B). Individual phages showed significant variation in their virulence across the three bacterial strains on both islands (Mauritius: *χ* ^2^ = 381.50, df = 10, *p* < 0.001; Reunion: *χ* ^2^ = 43.11, df = 9, *p* < 0.001), with significant phage–bacteria interactions indicating relative phage specialisation (Mauritius: *χ* ^2^ = 914.89, df = 20, *p* < 0.001; Reunion: *χ* ^2^ = 50.34, df = 18, *p* < 0.001). This result was unexpected on account of the genetic similarity of Reunion bacteria and the specialised host range of Reunion phages. As previously reported, we demonstrate that phage life cycle (virulent or temperate) was significantly linked with phage virulence (*χ* ^2^ = 49.29, df = 1, *p* < 0.001) (Figure S2) (Rollie et al. 2020). Most phages that have a weaker inhibitory effect on bacterial density are temperate.

### 3.6 In Environments With Higher Bacterial Genetic Diversity, Generalist Phages Are More Virulent: Specialists Thrive in Simpler Settings

**FIGURE 4:**
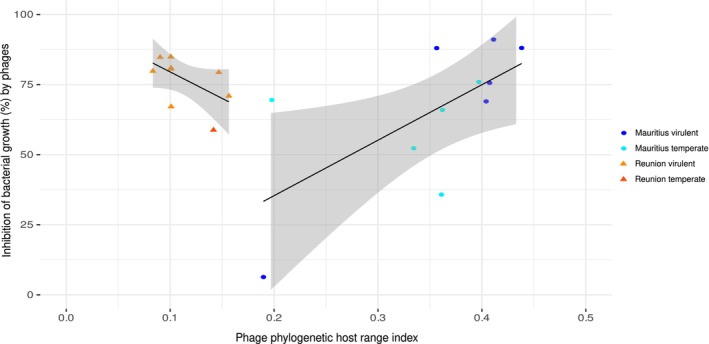
Correlation between maximum virulence, as the highest capacity of phages to inhibit bacterial growth, and the phylogenetic host‐range index of 21 phages, depending on the island of origin of phages. Datasets include 11 Mauritian phages confronted to 33 RSSC strains and 10 Reunion phages confronted to 20 RSSC strains. Data are average values, and the shaded areas represent the standard error.

We tested the hypothesis that generalist phages are less virulent than specialist ones, as a result of possible evolutionary trade‐offs between host range breadth and virulence (capacity to inhibit bacteria). No significant correlation between the PHRI and the maximum virulence value was observed for the whole data set. A significant positive correlation was detected for Mauritian phages (Spearman's *ρ* = 0.6818, df = 9, *p* = 0.0255) (Figure 4). This result implies that the most inhibitory phages were able to target more RSSC strains, with a higher genetic diversity. Also, it means that there is no apparent trade‐off for generalist phages on their capacity to reduce the fitness of their hosts. For Reunion phages, this trend was negative and significant, indicating that phage generalism comes at an evolutionary cost (lower virulence) in a homogeneous bacterial diversity situation (Spearman's *ρ* = −0.6727, df = 8, *p* = 0.0394) (Figure 4).

We also tested the association of phage virulence and host range with the rest of phage variables available (genome size, protein content, GC%, taxonomy, etc.). Most were non‐significant (Table S5), but phage life cycle (virulent or temperate) was associated with phage host‐range phylogenetic signal (*χ* ^2^ = 6.7108, df = 1, *p* = 0.0096) (Figure S3). It has been described before that temperate phages usually target closely related strains and have lower virulence (Moura de Sousa et al. 2021). Even with the small number of temperate phages (6 out of 23) and their high overall genetic diversity in our data set, we detect this particular significant trend. The phage genome GC% was positively correlated to the PHRI (Spearman's *ρ* = −0.4973, df = 23, *p* = 0.0158) (Figure S4). The average GC% of all 23 phages is lower than that of the bacterial host, which has a median GC% of 66.8, notably suggesting that phage host range expansion may be related to adaptation to the host codon usage. Also, phage load was significantly associated with their morphology, with highest loads for myovirus shapes (*χ* ^2^ = 9.2977, df = 2, *p* = 0.0096) (Figure S5), as previously shown (Kornienko et al. 2020).

Lastly, we investigated another crucial evolutionary trade‐off within host–parasite interactions by testing whether the range of hosts a parasite can infect has an impact on its overall infectious population size (phage load). Among phages, Pearson's coefficient of correlation between PHRI and phage load was *r* = −0.802 for Reunion and *r* = 0.205 for Mauritius phages (Figure S6). The *r* values obtained with random permutations of infectious population size values among phages ranged from −0.97 to 0.16 (mean −0.58) for Reunion and ranged from 0.021 to 0.395 (mean 0.208) for Mauritius ones. Out of the 1000 random permutations, 61 and 486 yielded lower *r* values than those calculated for the actual infectious population size and host‐range data of Reunion and Mauritius phages, respectively. Therefore, the correlation between phage load and host range did not depart significantly from correlations obtained with random data permutations for Mauritius phages. For Reunion phages, the correlation was only slightly more negative than what was expected under the null hypothesis defined by random permutations, with a *p* value of 0.061 (Figure S6). Consequently, only weak evidence of trade‐off between the infectious population size and host range was obtained for phages from Reunion Island. Variables such as counter‐defence mechanisms in phages and the characteristics of the receptor used may play a role in determining the breadth of host range and its impact on the viral population size. We hope further research and more genomic data from both bacteria and phages will shed light on this interesting topic.

## 4 Discussion

Understanding how phages specialise or generalise their host range is critical for both ecological theory and applied microbiology. Yet, assessing phage host range remains a challenge. While phylogenetic information has long been integrated into ecological studies, it is rarely applied in phage–bacteria systems (Gupta et al. 2022; Walsh et al. 2022). In contrast to previous studies, we used an ecology phylogenetic diversity index (PHRI) to determine the degree of generalism or specialisation of phages and employed enhanced matrix analysis to complement this approach. The consistency between PHRI values and matrix structure properties highlights the robustness of these methods. Compared to traditional metrics like EOP, which capture infection efficiency, and binary host counts (*N*), which reflect the number of susceptible hosts, neither accounts for the phylogenetic relationships among those hosts. This is particularly valuable when phages infect closely related strains with varying efficiencies, where EOP may underestimate breadth and *N* may overestimate it by including weak or marginal infections. PHRI integrates all three dimensions—host number, infection strength and phylogenetic breadth—providing a more balanced and biologically relevant measure for comparing phage generalism and specialisation. Although these methods are reliable on their own, integrating phage phylogeny into host‐range studies presents an additional challenge, as phages often lack sufficient genetic homology to establish meaningful evolutionary relationships.

Depending on the genetic, evolutionary and mechanistic patterns of host–parasite interactions, contrasted scores for nestedness and modularity are expected. Modularity has been frequently observed when the evolutionary or ecological scales considered for bacteria and/or phages are wide (i.e., broad time frames or highly diverse habitats) (Kauffman et al. 2022; Piel et al. 2022). Recent work elegantly demonstrates that even in closely related bacterial populations, modules can emerge over short timescales due to phage‐bacteria co‐evolutionary dynamics (Borin et al. 2023). Nested networks range from broad to narrow host ranges, but the degree of generalism depends also on bacterial diversity. In staphylococcal phages, for instance, previous research revealed a predominantly nested interaction structure, with some phages successfully infecting bacterial hosts across multiple species (Göller et al. 2021).

In our study, Reunion phages exhibited a narrow host range, predominantly infecting closely related RSSC strains, leading to lower PHRI values. In contrast, Mauritian phages displayed an extremely diverse host‐range spectrum, ranging from extreme specialists to broad generalists within defined interaction modules. The larger host‐range breadth and diversity of Mauritian phages is likely associated with the more complex genetic diversity of RSSC in the island, with a previously calculated Simpson's Diversity Index of 0.907 and a genetic richness of 16.35, compared to the 0.485 and 5.41 values of RSSC bacteria isolated from Reunion (Cellier et al. 2023). This pattern can be partially explained by different agricultural practices. In Mauritius, local potato and tomato breeding practices, along with high cultivar diversity, may facilitate RSSC strain exchanges and foster bacterial genetic diversity. Reunion primarily relies on certified seed imports from Metropolitan France, potentially limiting bacterial strain diversity. Other particularities of the sampling setting were that our focus was on the agriculturally rich, low‐altitude regions of the island, where phylotype I is more prevalent and to which phages are shown to be adapted. At high altitude areas of Reunion, more phages adapted to phylotype II, and possibly III, would probably have been found, because of the lower temperature optimum and greater prevalence of these RSSC variants in potato crops (Scherf et al. 2010). This spatial compartmentalisation does not exist in Mauritius. All in all, the collected phages may be a good proxy of the main phage diversity present in the major agricultural zones of both islands frequently affected by bacterial wilt (Trotereau et al. 2021).

Positive correlations between host range and virulence have been proven in other organisms such as plant‐feeding insects or various plant parasites (Moury et al. 2021; Peterson et al. 2015). The links between these two traits in phages have been poorly explored, but we show that they depend on bacterial diversity. Specialist phages perform better in homogeneous settings, while heterogeneous environments may promote phage niche expansion without significant costs, as supported by meta‐analyses of microbial evolution experiments (e.g., Bono et al. 2017; Kassen 2002). We suggest that Mauritian phages may have optimised their replication and host inhibitory capacity as a consequence or as a prerequisite for their expanded host range. Reunion phages display a contrasted situation, where co‐evolution or epidemiological dynamics between phages and bacteria in this island have selected for specialist phages. When the genetic landscape simplifies, specialists emerge as the most efficient phage type, presenting a fine‐tuned adaptation to specific host environments. However, phages could exhibit different virulence levels if other RSSC variants were tested. Even if our study was limited to two locations only and we targeted a specific bacterial species complex, our findings suggest that the host range versus virulence evolutionary trade‐off may not be universal in phages. Instead, positive associations between these traits may be frequent in environments with higher genetic diversity. This conveys great advantages for generalist phage populations in nature and keys for the selection of candidate phages in therapeutic or biocontrol applied settings. Still, much needs to be tested, as mutations that facilitate phage generalism and virulence may, in turn, lead to diminished fitness in different environments or evolutionary time scales (Bono et al. 2020; Chevallereau et al. 2022; Sant et al. 2021).

Thanks to in‐depth monitoring of RSSC in the SWIO islands, phylogenetic and epidemiological data are available, and we could link phage host range to bacterial diversity. Our work relies on bacterial taxonomy, primarily focusing on a single gene that allows us to reconstruct the phylogeny of the studied bacteria. We questioned whether similar methods involving all available genes present in the core genome of bacteria would produce analogous results. Our study is essential alongside research focusing on mechanisms, which will help identify genetic factors influencing phage host range in bacteria. For example, comparative genomics of distantly related bacteria targeted by the same phage, which likely share receptors (e.g., phylotypes IV and I or II of RSSC), can help in uncovering these genetic determinants (Gaborieau et al. 2024; Maffei et al. 2021).

A previous study showed that intact prophages are found in 88% of 192 published RSSC genomes, and that individual prophages are restrained to 2–3 continents and are phylotype‐specific (Greenrod et al. 2022). Few exceptions are highlighted, one being phage Dina from Mauritius, a temperate phage with a high PHRI (generalist), which is distributed and abundant in multiple phylotypes from all six continents (Greenrod et al. 2022). Although the other five temperate phages studied here were not detected in Greenrod et al. (2022), this result aligns with PHRI data, supporting the idea that generalism in Mauritian phages is an effective evolutionary strategy for widespread infection.

One limitation of this study is the use of a CRISPR‐Cas spacer database that included only *R. solanacearum* genomes, excluding the other two major species of the RSSC. Additionally, the database was relatively limited in size and not recently updated (Dion et al. 2021). Existing CRISPR‐Cas analysis tools were also not well adapted to the specific questions we sought to explore (Biswas et al. 2016; Motoche‐Monar et al. 2024). Improving the coverage and quality of these resources would be instrumental in better elucidating the potential links between CRISPR‐Cas systems and phage–host range interactions (PHRI). Whether CRISPR‐Cas systems *preferentially* target generalists remains unclear. It is possible that generalists are simply more likely to be detected due to more frequent host encounters, rather than due to increased CRISPR effectiveness. Alternatively, a high viral load (i.e., phage fitness) may play a larger role in spacer acquisition than host range breadth alone. We hope that future studies will investigate the nature and prevalence of prophages, CRISPR‐Cas systems, as well as other defence systems, in the strains used here.

Our approach provides a scalable framework for studying phage–bacteria interactions across diverse ecosystems. Rooted in classic community ecology, the PHRI approach demonstrates how ecological concepts can enhance our understanding of phage interactions. From an applied perspective, our results suggest that matching the choice of phage cocktails to the ecological context could enhance efficacy: specialist phages may be more effective in controlled, low‐diversity settings (e.g., greenhouse crops or hospital infections dominated by a single clone), whereas heterogeneous environments–such as complex soil microbiomes or polymicrobial infections–may favour the use or evolution of broad‐host‐range phages without compromising performance. By linking phage host range to bacterial diversity, our study sheds light on phage–bacteria co‐evolution and identifies key factors for optimising phage‐based strategies in agricultural and clinical applications.

## Author Contributions

C.T.‐B. designed research; C.T.‐B. and C.B. performed the laboratory work; C.T.‐B., J.R.G. and B.M. analyzed data; I.R. and S.P. provided materials; C.T.‐B. wrote the paper; all authors provided editorial inputs and approved the final version of the manuscript.

## Disclosure

Benefit‐Sharing Statement: Benefits from this research accrue from the sharing of our data and results on public databases as described above. Also, a research collaboration was developed with scientists from the countries providing genetic samples, included as co‐authors in a previous publication.

## Conflicts of Interest

The authors declare no conflicts of interest.

## Supporting information


**Appendix S1:** Supplementary methods.
**Figure S1:** Correlations between bacteria resistance spectrum and average sensitivity to phages.
**Figure S2:** Relationship between phage maximum inhibitory effect on bacterial density and phage life.
**Figure S3:** Relationship between phage host‐range phylogenetic signal and phage life cycle (temperate).
**Figure S4:** mec70052‐sup‐0001‐AppendixS1.pdf. Relationship between phylogenetic host‐range index relationship and the GC% genome.
**Figure S5:** Relationship between phage load and morphology for a set 23 phages.
**Figure S6:** Correlation (Pearson's *r*) between phage host range and infectious population size.

**Table S1:** List of *Ralstonia solanacearum* strains used for assessing the host range of phages from Mauritius and Reunion islands. Ii includes phylogenetic assignment, year and location of isolation, along with the corresponding *egl* sequence used for sequevar classification.

**Table S2:** Genomic content comparison of six phage pairs, illustrating the quantity and type of shared and unique proteins (highlighted in orange), alongside coding gene lengths and positions.

**Table S3:** Summary statistics for pairs of phage variables. We used non‐parametric Kruskal–Wallis tests for factor variables and Spearman correlation tests for numeric variables. *p*‐values in bold indicate statistically‐significant tests and have been illustrated in figures. *NA* indicates impossible tests due to spurious correlations that have been analysed accordingly.

**Table S4:** Comparison of conventional host range metrics with the phage phylogenetic host rangeindex (PHRI). For the Reunion phage‐bacteria dataset, we calculated the efficiency of plating(EOP), and number of hosts with EOP < 0.1, and compare these values to the PHRI.

Table S4:

## Data Availability

The data supporting the results can be found here https://doi.org/10.5061/dryad.tmpg4f596.
