# Supplementary material for: A Phylogenetic Host‐Range Index Reveals Ecological Constraints in Phage Specialisation and Virulence
Source: Mol Ecol. 2025 Jul 28;34(18):e70052. doi: 10.1111/mec.70052 (PMC12421481; doi:10.1111/mec.70052)
Supplement: Supplementary file 1 — Appendix S1: Supplementary methods. Figure S1: Correlations between bacteria resistance spectrum and average sensitivity to phages. Figure S2: Relationship between phage maximum inhibitory effect on bacterial density and phage life. Figure S3: Relationship between phage host‐range phylogenetic signal and phage life cycle (temperate). Figure S4: mec70052‐sup‐0001‐AppendixS1.pdf. Relationship between phylogenetic host‐range index relationship and the GC% genome. Figure S5: Relationship between phage load and morphology for a set 23 phages. Figure S6: Correlation (Pearson's r) between phage host range and infectious population size. [file MEC-34-e70052-s005.pdf]

SUPPLEMENTARY FILE FOR:

A phylogenetic host-range index reveals ecological constraints in phage specialisation and virulence

Clara Torres-Barceló[a#], Claudine Boyer[b], Julian R. Garneau[c], Stéphane Poussier[d], Isabelle Robène[b] and Benoît Moury[e]

a PHIM Plant Health Institute, Univ Montpellier, INRAE, CIRAD, Institut Agro, IRD, Montpellier, France

b CIRAD, UMR PVBMT, F-97410 St Pierre, La Réunion, France

c Department of Fundamental Microbiology, University of Lausanne, CH-1015 Lausanne, Switzerland

d Université de la Réunion, UMR PVBMT, F-97410 St Pierre, La Réunion, France

e INRAE, Pathologie Végétale, F-84140, Montfavet, France

clara.torresbarcelo@inrae.fr

List of content:

Supplementary Methods

Legends to Tables S1, S2, S3, S4 and S5

Figures S1 to S6.

Supplementary References

Supplementary Methods

Methods to estimate nestedness and modularity are described in Weitz et al. [1]. Two algorithms were available to estimate nestedness of matrices with quantitative data: the weighted nestedness metric based on overlap and decreasing filling (wNODF algorithm) [2] and the weighted-interaction nestedness estimator (WINE algorithm) [3]. In the R software, the 'nested' and 'wine' functions were used to estimate the wNODF and WINE scores, respectively. We used six different algorithms implemented into R software to estimate modularity: the spinglass [4–6], edge betweenness [4], fast greedy [7], leading eigenvector [8], louvain [9] and DIRTLPAwb+ [10] algorithms. We also used the QuanBiMo algorithm [11] to estimate the modularity score but not to estimate its statistical significance, since it was excessively slow to analyse simulated datasets and since it provided modularity scores identical to DIRTLPAwb+. Moreover, DIRTLPAwb+ was shown to consistently match or outperform QuanBiMo [10]. Additional algorithms (label prop and walktrap) did not allow correct estimations of modularity (data not shown).

To determine the statistical significance of the nestedness or modularity of the phage-bacteria interaction matrices, the nestedness/modularity scores of the actual matrices derived from experimental data were compared to those of simulated null-model matrices that are not expected to possess any nested or modular pattern. Actual matrices were compared to matrices simulated under seven different null models [12]. Nestedness (or modularity) is significant if the actual matrix is more nested (or modular) than at least 95% of the matrices simulated under a given null model. Moury et al. [12] compared the performance (type I and type II error rates) of the nestedness and modularity algorithms and associated null models (Supplementary Methods 2 in Moury et al. 2021). For nestedness, a higher statistical power was observed for WINE than wNODF algorithm, whatever the null model. Consequently, we focused mainly on the results of the WINE algorithm.

For modularity, the spinglass algorithm was by far the most efficient in terms of type I error rate, whatever the null model. For four other modularity algorithms included in [10] (*i.e.* edge betweenness, fast greedy, leading eigenvector and Louvain), the type I error rate varied greatly depending on the null model. Also, null models C1, R1, C2 and R2 were the most suitable for both nestedness and modularity in terms of performance. Indeed, matrices showing significant patterns of nestedness or modularity with both models C1 and R1 (or both models C2 and R2) had the lowest type I error rates [10]. Finally, we did not evaluate the performance of modularity algorithm DIRTLPAwb+ in our earlier study [12] but its results with the Mauritius and Reunion matrices were very close to the results obtained with algorithm fast greedy in terms of modularity scores, significance values and effect sizes, whatever the null model.

Table legends

Table S1: List of *Ralstonia solanacearum* strains used for assessing the host range of phages from Mauritius and Reunion islands. Ii includes phylogenetic assignment, year and location of isolation, along with the corresponding *egl* sequence used for sequevar classification.

Table S2: Genomic content comparison of six phage pairs, illustrating the quantity and type of shared and unique proteins (highlighted in orange), alongside coding gene lengths and positions.

Table S3: List of CRISPR spacers (N=233) identified in 20 *R. solanacearum* genomes that match 22 phages from the Mauritius and Reunion collection, allowing up to two mismatches.

Table S4: Comparison of conventional host range metrics with the phage phylogenetic host range index (PHRI). For the Reunion phage-bacteria dataset, we calculated the efficiency of plating (EOP), and number of hosts with EOP > 0.1, and compare these values to the PHRI.

Table S5: Summary statistics for pairs of phage variables. We used non-parametric Kruskal-Wallis tests for factor variables and Spearman correlation tests for numeric variables. P-values in bold indicate statistically- significant tests and have been illustrated in figures. *NA* indicates impossible tests due to spurious correlations that have been analysed accordingly.

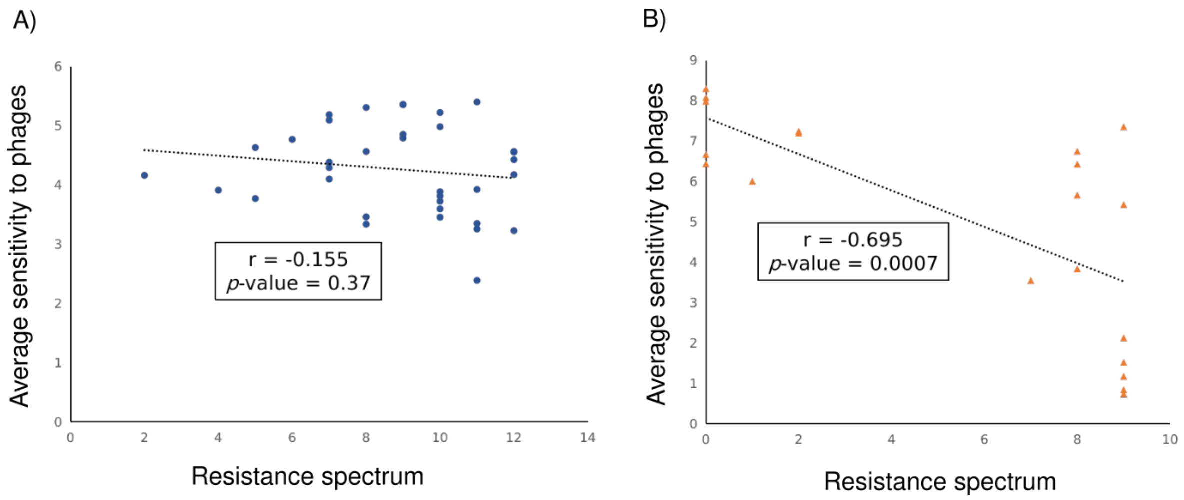

Figure S1: Correlations between bacteria resistance spectrum and average sensitivity to phages. Datasets include 36 bacteria and 13 phages for Mauritius (A) and 20 bacteria and ten phages for Reunion (B).

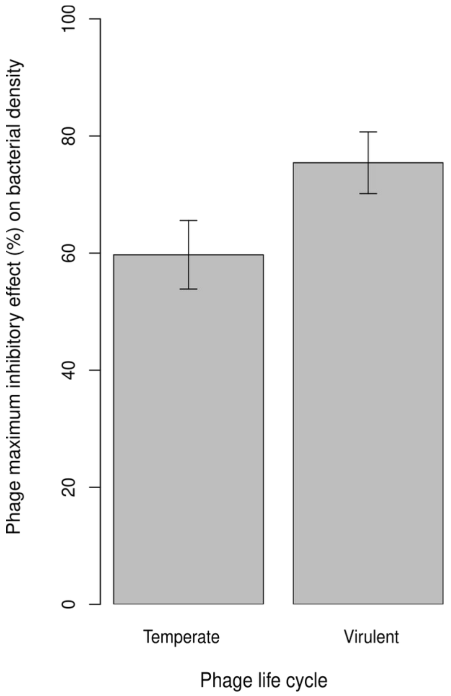

Figure S2: Relationship between phage maximum inhibitory effect on bacterial density and phage life cycle (temperate or virulent) for a set of 21 phages.

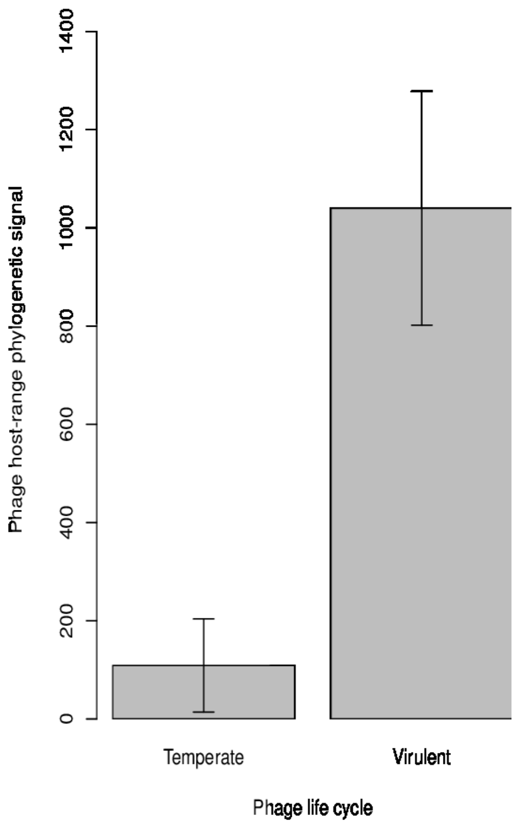

Figure S3: Relationship between phage host-range phylogenetic signal and phage life cycle (temperate or virulent) of 23 phages.

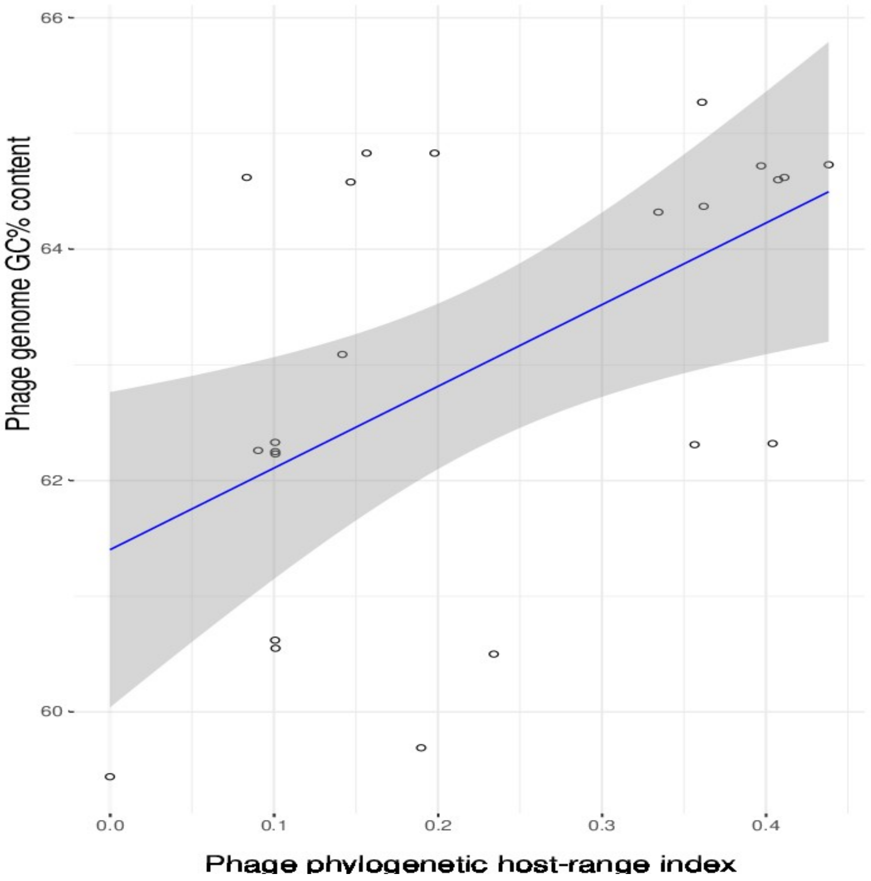

Figure S4: Relationship between phylogenetic host-range index relationship and the GC% genome content inhibitory effect in 23 phages.

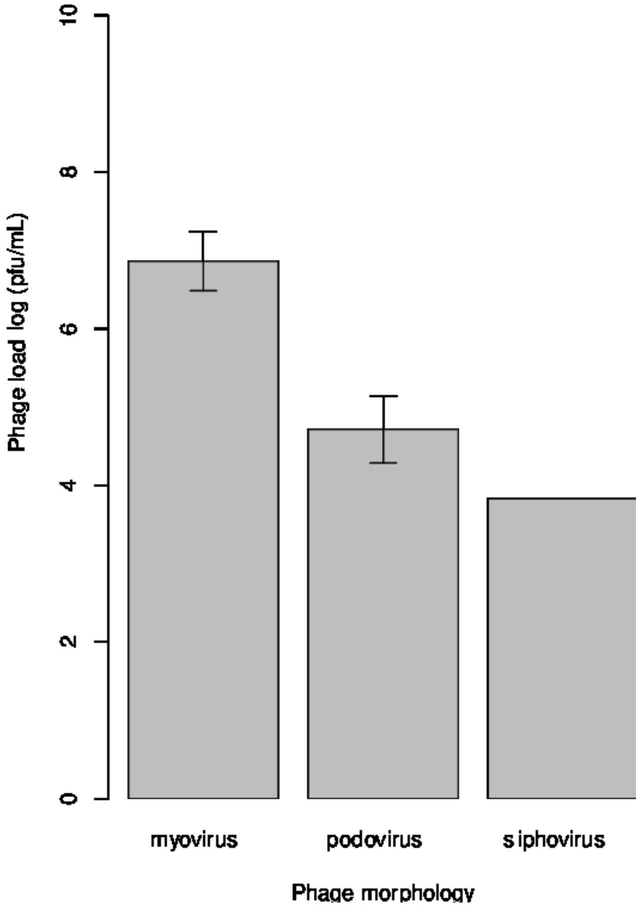

Figure S5: Relationship between phage load and morphology for a set 23 phages.

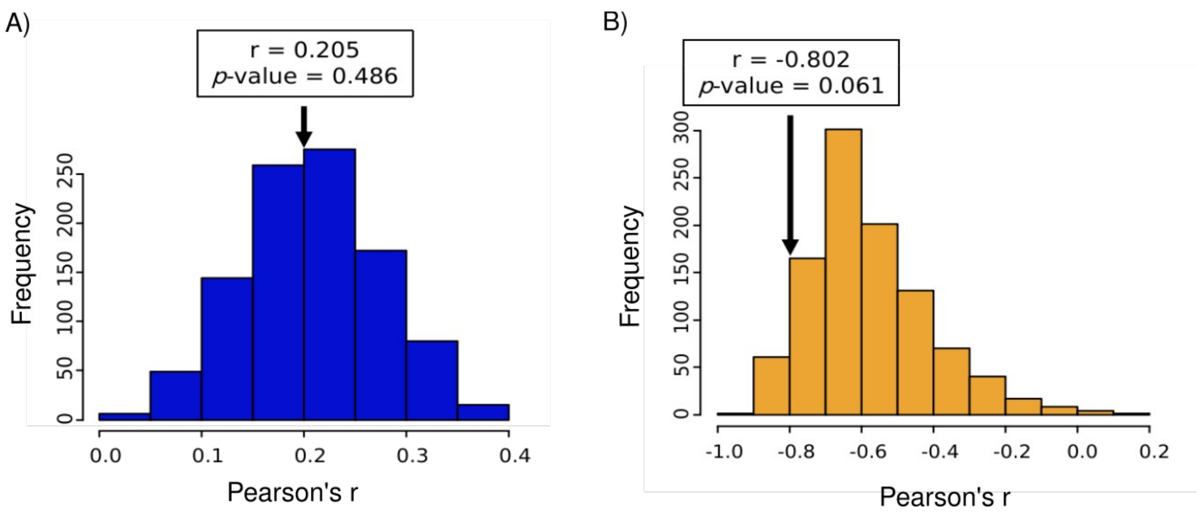

Figure S6: Correlation (Pearson's r) between phage host range and infectious population size for Mauritius (A) and Reunion (B) datasets. The r values of actual datasets are indicated by arrows. Because infectious population sizes were used to calculate host range (PHRI), these two variables are not independent and there is a risk of "spurious correlation". As a consequence, r=0 is not a proper null hypothesis. Instead, we determined *ad hoc* null distributions of r values with 1,000 random permutations of infectious population sizes across bacterial strains for each phage (blue and orange distributions). The percent of random permutations showing lower r values than that of the actual dataset provides a p-value for the significance of the correlation.

Supplementary References

1. Weitz JS, Poisot T, Meyer JR, Flores CO, Valverde S, Sullivan MB, et al. Phage–bacteria infection networks. *Trends in Microbiology* 2013; **21**: 82–91.
2. Almeida-Neto M, Guimarães P, Guimarães Jr PR, Loyola RD, Ulrich W. A consistent metric for nestedness analysis in ecological systems: reconciling concept and measurement. *Oikos* 2008; **117**: 1227–1239.
3. Galeano J, Pastor JM, Iriondo JM. Weighted-Interaction Nestedness Estimator (WINE): A new estimator to calculate over frequency matrices. *Environmental Modelling & Software* 2009; **24**: 1342–1346.
4. Newman MEJ, Girvan M. Finding and evaluating community structure in networks. *Phys Rev E* 2004; **69**: 026113.
5. Reichardt J, Bornholdt S. Statistical mechanics of community detection. *Phys Rev E* 2006; **74**: 016110.
6. Traag VA, Bruggeman J. Community detection in networks with positive and negative links. *Phys Rev E* 2009; **80**: 036115.
7. Clauset A, Newman MEJ, Moore C. Finding community structure in very large networks. *Phys Rev E* 2004; **70**: 066111.
8. Newman MEJ. Finding community structure in networks using the eigenvectors of matrices. *Phys Rev E* 2006; **74**: 036104.
9. Blondel VD, Guillaume J-L, Lambiotte R, Lefebvre E. Fast unfolding of communities in large networks. *J Stat Mech* 2008; **2008**: P10008.
10. Beckett SJ. Improved community detection in weighted bipartite networks. *Royal Society Open Science* 2016; **3**: 140536.
11. Dormann CF, Strauss R. A method for detecting modules in quantitative bipartite networks. *Methods in Ecology and Evolution* 2014; **5**: 90–98.
12. Moury B, Audergon J-M, Baudracco-Arnas S, Ben Krima S, Bertrand F, Boissot N, et al. The quasi-universality of nestedness in the structure of quantitative plant-parasite interactions. *Peer Community Journal* 2021; **1**: e44.
